# Prejudice and determinants regarding tuberculosis patients among medical students in Dalian, Northeast China: a cross-sectional study

**DOI:** 10.3389/fpubh.2023.1292333

**Published:** 2024-01-10

**Authors:** Yaohui Yi, Meng Sun, Xu Chen, Yuanping Pan, Jiachen Lu, Yingying Yu, Xiaofeng Dou, Ling Zhou

**Affiliations:** School of Public Health, Dalian Medical University, Dalian, Liaoning, China

**Keywords:** tuberculosis, prejudice, student, associated factors, China

## Abstract

**Background:**

Health workers play a central role in global tuberculosis (TB) elimination efforts. If medical students have prejudiced attitudes toward TB patients, this may make it difficult for them to provide effective health care to TB patients in their future roles as health workers. There is currently no research on prejudice toward TB patients among medical students in China. This study aimed to explore the current status of medical students’ prejudice against patients with TB and its associated predictors.

**Methods:**

We conducted a cross-sectional questionnaire survey among medical students at a medical university in Dalian, Liaoning Province, Northeast China. Multiple logistic regression analysis was employed to determine the predictive factors of medical students’ prejudice against patients with TB.

**Results:**

More than half (57.23%) of the medical students held prejudices against individuals with TB. Multivariate logistic regression analysis revealed that not receiving TB health education (OR: 2.12, 95% CI: 1.35–3.32), not knowing a person with TB (OR: 2.52, 95% CI: 1.39–4.56), and fear of TB/TB patients (OR: 6.79, 95% CI: 4.36–10.56) were identified as risk factors for medical students’ prejudice against TB patients. Conversely, residing in rural areas (OR: 0.60, 95% CI: 0.38–0.95), agreeableness (OR: 0.82, 95% CI: 0.73–0.92) and emotional stability (OR: 0.90, 95% CI: 0.81–1.00) in the Big Five personality traits, and a better understanding of TB knowledge (OR: 0.58, 95% CI: 0.38–0.89) were identified as protective factors.

**Conclusion:**

In China, a considerable number of medical students still exhibit prejudice against patients with TB. Targeted interventions, such as incorporating TB health education into the core curriculum of medical students, and enhance their agreeableness and emotional stability, are still needed. Furthermore, greater focus should be placed on medical students from urban backgrounds or those who harbor fear or do not know a person with TB.

## 1 Introduction

Tuberculosis (TB) remains the second leading cause of death globally from a single infectious agent, following COVID-19, and causes nearly twice as many deaths as HIV/AIDS (1). It has been recognized as a global public health emergency for the past 25 years and has been named one of the six infectious diseases that threaten the world’s population (2–4). The World Health Organization (WHO) established a global target to eradicate TB by 2035. According to the WHO, China would continue to carry a substantial burden of TB in 2020, trailing only India, with 8.5% of the world’s TB cases. China’s future preventive and control situation is still dire to accomplish this goal (5, 6). Along with the need to concentrate on treatment and cure rates, a more holistic approach to disease control is now being promoted in order to address poverty, stigma, and other social risk factors, of which stigma, which is a major barrier to the global elimination of TB (7, 8), because it delays patient diagnosis and treatment. Studies in different backgrounds report that about 42–82% of TB patients suffer from stigma, while studies from China show that 45.32% of TB patients suffer from stigma (9–11). Reducing stigma is crucial for TB control.

Prejudice is an inadequate judgment used to define something or someone and establish an idea without prior knowledge and characterized by the affirmation of own identity as superior/dominant, and likewise, by the denial of the other who is different (12). Prejudice is the basis of stigma, which leads society to have negative attitudes toward infectious diseases and their victims and to isolate them (13). Fear-based policies (e.g., pre-emptive isolation, involuntary testing, etc.) have normalized biased perceptions of TB patients among health professionals and society at large by highlighting the serious threat that TB poses to the public, this has increased the burden of fighting TB, particularly in countries with a high TB burden, and the growing recognition that this approach is both ethically and scientifically indefensible (8, 14). Furthermore, when health workers have negative attitudes toward people with TB, it can lead to increased stigma against the disease throughout society, with wider socio-economic implications (15, 16). Perhaps there may be considerable ambivalence in the minds of health workers who, despite trying to help TB patients, retain prejudices and treat TB patients in a discriminatory manner, which can have a considerable impact on TB patients (17). The social manifestations of TB patients indicate that they are afraid of stigma due to prejudice among health workers and the public, and therefore act in secrecy about the disease (18, 19). Medical students, as the reserve force of health workers, may come into contact with TB patients in the future, and their attitude toward patients is also related to the treatment results of patients. It is crucial to lessen medical students’ prejudice toward TB patients, and attention should be paid to the problem of medical students’ prejudice toward TB patients as well as the creation of interventions.

A small number of studies have been conducted to determine the factors associated with prejudice against people with TB. Several socio-demographic characteristics, such as age, gender, socio-economic status, differences in TB health education and educational environment among medical schools, as well as beliefs and lack of knowledge about TB transmission, are important factors influencing prejudice against TB patients (20–25).

To the best of our knowledge, in Dalian, Liaoning province, China, there have been no studies on prejudice toward TB patients among medical students have been conducted. In this study, we aimed to understand the factors associated with medical students’ prejudice toward TB patients, to effectively reduce the prejudice of these future health workers toward TB patients.

## 2 Materials and methods

### 2.1 Study area, study design and study period

Dalian is an economically developed coastal city in Liaoning Province, Northeast China, covering an area of 12,574 square kilometers, and is an important port, industrial, trade, financial, and tourist city in China. The *per capita* gross domestic product (GDP) of Dalian was 113,200 yuan in 2022, ranking first in Liaoning Province, and the city’s household population was 6,087,000 as of the end of 2022. According to the statutory report of Liaoning Province, the incidence rate of TB in 2018 was 55.81/100,000, and the mortality rate was 0.49/100,000 ranking fourth in the country (26). In addition, according to a recent study, more than half of TB patients in Dalian hold a sense of disease shame due to differential treatment by health workers as well as family and friends (27). As future health workers, medical students’ attitudes toward TB patients may also influence public perceptions of patients. Therefore, we conducted a cross-sectional survey of medical students from March 2022–June 2022 at the only public comprehensive medical university in Dalian.

### 2.2 Participants

Most studies in China tend to define undergraduate medical students as undergraduate students enrolled in an undergraduate medical school and majoring in a medical-related specialty (28–33). The subjects of the study were first- to fourth-year medical undergraduates from various medical specialties at Dalian Medical University. During the study period, there were 7,916 medical students in their first to fourth year of university, including the Department of Clinical Medicine, Department of Forensic Medicine, Department of Anesthesiology, Department of Medical Laboratory Science, Department of Nursing, Department of Health Management, Department of Basic Medical Sciences, Department of Preventive Medicine, Department of Medical Imaging, Department of Clinical Medicine of Chinese and Western Medicine, and Department of Stomatology (34, 35). University faculty and staff, medical students who had graduated with an undergraduate degree, non-medical students, and medical students who did not sign informed consent were excluded from this study. High school graduates from all over China were admitted only if they passed the entrance examination and met the admission criteria of Dalian Medical University.

### 2.3 Sample size and sampling technique

Since there is no similar study in the target group in our study area, the sample size was determined by a single proportion formula using (36): *N* = *Z*^2^*P(1–*P*)/*d*^2^; where *Z* = 1.96, *p* = 50%, *d* = 0.05, accordingly the sample size will be 384. Since the total population of medical students on campus in their first to the fourth year of university is 7,916, adjustment was made by the following formula nf = ni/[1 + (ni/N)] (37); where nf is the sample size calculated by adjustment, ni is the initial sample size calculated by single proportion formula (384), N is the total number of students in the campus (7916). Based on this formula the sample size for this study was 366. The sample size was increased by 50% to minimize the effect of sampling error. A simple random sampling technique was used to distribute the 549 paper questionnaires proportionally to medical students of all years. The final number of valid questionnaires returned was 505, with a return rate of 92%.

### 2.4 Data collection

The data was collected through a structured questionnaire designed by the researcher. The questionnaire was designed based on extensive reference to relevant literature and consultation with experts in the field. To ensure that respondents accurately understood the meaning of each item in the questionnaire, a pre-survey was conducted at the study site and the questionnaire was modified accordingly. Five categories made up the questionnaire: socio-demographic characteristics, Big Five personality, knowledge of TB, fear and prejudice toward people with TB. Sociodemographic characteristics included gender, age, place of residence, grade level, whether they had received health education about TB and whether they know a person with TB. All participants signed an informed consent form.

#### 2.4.1 Scale for prejudice against people with TB

The scale to measure prejudice against TB patients was designed with reference to TB stigma measurement guidelines and related literature (38). The scale demonstrated adequate reliability (Cronbach’s *α* = 0.87, KMO = 0.84) and results from a one-factor confirmatory factor analysis demonstrated a good fit (x2/df = 5.079, NFI = 0.983, AGFI = 0.933, IFI = 0.962, CFI = 0.986, TLI = 0.965, RMR = 0.026) (39). Each item on the scale has six response options:1 (strongly disagree), 2 (moderately disagree), 3 (somewhat disagree), 4 (somewhat agree), 5 (moderately agree), and 6 (strongly agree). The total score ranges from 6 to 36, with higher scores reflecting more severe prejudice. As for the judging criteria of the scale cut-off value, this scale refers to the study of Jittimanee SX et al., the median score of the study population was used as the cut-off value for the scale (40). Respondents with a total personal prejudice score greater than or equal to the median score were classified as prejudiced against TB patients, while respondents with a total personal prejudice score below the median score were classified as non-prejudiced against TB patients.

#### 2.4.2 Fear of TB/TB patients scale

We developed a 4-item measure to assess medical students’ fear of TB/TB patients. The scale has good reliability (Cronbach’s *α* = 0.71, KMO = 0.69) and results from a one-factor confirmatory factor analysis demonstrated a good fit (x2/df = 2.018, NFI = 0.995, AGFI = 0.980, IFI = 0.997, CFI = 0.997, TLI = 0.984, RMR = 0.005). Each item on the scale has four response options:1 (strongly disagree), 2 (disagree), 3 (agree) and 4 (strongly agree). The total score ranges from 4 to 16, with higher scores reflecting more severe fears. This scale refers to the study of Jittimanee SX et al., the median score of the study population was used as the cut-off value for the scale (40). Respondents with a total personal fear score greater than or equal to the median score were classified as fearful of TB/TB patients, while respondents with a total personal fear score below the median score were classified as non-fearful of TB/TB patients.

#### 2.4.3 Ten-item personality inventory (TIPI-C)

Several scales have been developed and validated to measure personality traits (41). Nevertheless, due to a large number of entries, it takes a long time to accurately measure personality (42).To lighten the load of the response, several shorter instruments have been developed, such as the 44-item Big Five Inventory (BFI), the 60-item New Five-Factor Inventory (NEO-FFI) and the 100 Trait Descriptive Adjectives(TDA) (41, 43). The BFI, NEO-FFI and TDA have been reported to take approximately 5, 15 and 15 min to complete, and these instruments are still considered too long for use in large-scale population surveys, in addition to the fact that medical students are often under pressure to study and have limited time, making the use of the shortest Big Five Personality Inventory (TIPI-C) more appropriate for this study (44–46). The scale was developed with Chinese socio-cultural characteristics in mind and contains 10 items with 5 dimensions: agreeableness, conscientiousness, extraversion, openness and emotional stability, when calculating the dimensional scores (i.e., the average score of two items), one of the items needs to be reverse coded (e.g., on a 7-point scale, the score changes from “7” to “1” and vice versa) (47). Reverse-coded items are 2, 4, 6, 8 and 10, with higher scores indicating higher levels of testing. Previous studies have shown that the intrinsic reliability of the scale is less optimistic, probably due to the small number of entries for each dimension, but it has good retest reliability and validity and still has value for application (48).

#### 2.4.4 Knowledge of TB

TB knowledge is mainly aimed at transmission and consists of the following five questions: (I) How is TB transmitted? (II) what is the main route of transmission of TB (III) Can all TB patients be transmitted to others? (IV) How long can the infectivity of ordinary pulmonary TB patients be significantly weakened or disappeared after general treatment? (V) TB can generally be spread through the patient’s clothing and blankets. Get 1 point for each correct answer. Respondents with a total personal knowledge score greater than or equal to the median score were classified as having good knowledge of TB transmission, while those with a total personal knowledge score below the median score were classified as having poor knowledge of TB transmission (Table 1).

**Table 1:** Knowledge about TB among medical students.

Question	Responses	*n*	%
How is TB transmitted?	Correct	425	84.16
	Incorrect	42	8.32
	Do not know	38	7.52
What is the main route of transmission of TB	Correct	287	56.83
	Incorrect	181	35.84
	Do not know	37	7.33
Can all TB patients be transmitted to others?	Correct	386	76.44
	Incorrect	42	8.32
	Do not know	77	15.25
How long can the infectivity of ordinary pulmonary TB patients be significantly weakened or disappeared after general treatment?	Correct	14	2.77
	Incorrect	240	47.52
	Do not know	251	49.70
TB can generally be spread through a patient’s clothing and blankets.	Correct	195	38.61
	Incorrect	137	27.13
	Do not know	173	34.26

TB: Tuberculosis infection.

### 2.5 Statistical analysis

After the questionnaire, which was collected on-site, was checked for completeness and correctness, the original data were entered into a database in EpiData 3.1 (EpiData Association, Odense, Denmark) software using double entry, and consistency was assessed, then exported to Stata version 16.0 (Stata Corp LLC, College Station, Texas, USA) and AMOS16.0 for analysis. Shapiro–Wilk was first used to test the normality of the data studied, and the results showed that the data studied did not follow a normal distribution, so we analysed the median and quartiles of the continuous data and the frequencies and percentages of the categorical data. Continuous variables were compared using the rank sum test to assess differences between the two groups. Categorical variables were compared using the chi-square test and Fisher’s exact test to assess differences between the two groups. Variables with *p*-values<0.10 were incorporated into a multiple logistic regression analysis, correcting for confounding factors while ensuring that more valuable information was included in the model to identify factors that contribute to medical students’ prejudice against TB patients. All comparisons were bilateral, and *p*-values<0.05 was considered to be statistically significant in multivariate logistic regression.

## 3 Results

### 3.1 Sociodemographic characteristics and their relationship to TB prejudice

The average age of the 505 medical students participating in the study was 20.07 ± 1.50 years, more than half of the medical students (60.40%) were female, the majority of medical students (70.10%) were from urban areas, the largest proportion was in their fourth year of university (26.14%), nearly two-thirds of the medical students (61.78%) had received health education on TB, and most medical students (84.55%) do not know a person with TB. More than half of medical students (57.23%) have a prejudice against people with TB. The univariate analysis showed that medical students whose families lived in urban areas and who did not know a person with TB were more likely to be prejudiced against people with TB, and those who had received health education on TB were less likely to be prejudiced against people with TB (All *p*-values<0.1) (Table 2).

**Table 2:** Sociodemographic characteristics of the medical students and their relationships with TB-related prejudice.

Variable	Student		Prejudice against TB patients		*P*
	*N*	%	Yes (%)	No (%)	
Sex					0.789
Male	200	39.60	113 (56.50)	87 (43.50)	
Female	305	60.40	176 (57.70)	129 (42.30)	
Age					0.586
17–20	288	57.03	168 (58.33)	120 (41.67)	
21and above	217	42.97	121 (55.76)	96 (44.24)	
Place of residence					**0.002**
Urban area	354	70.10	218 (61.58)	136 (38.42)	
Rural area	151	29.90	71 (47.02)	80 (52.98)	
Grade					0.834
Freshman	126	24.95	72 (57.14)	54 (42.86)	
Sophomore	122	24.16	74 (60.66)	48 (39.34)	
Junior	125	24.75	70 (56.00)	55 (44.00)	
Senior	132	26.14	73 (55.30)	59 (44.70)	
Whether you received TB health education?					**<0.001**
Yes	312	61.78	152 (48.72)	160 (51.28)	
No	193	38.22	137 (70.98)	56 (29.02)	
Know a person with TB?					**<0.001**
Yes	78	15.45	25 (32.05)	53 (67.95)	
No	427	84.55	264 (61.83)	163 (38.17)	

TB: Tuberculosis;Some *p*-values are highlighted in bold because they are less than 0.1.

### 3.2 The relationship between Big Five personality and TB prejudice

Since the data did not follow a normal distribution, we utilized medians and quartiles to describe the Big Five personality scores of medical students with and without prejudice against TB patients. For extraversion, the median score was 4.00, with a range of 3.00–5.00 (25th to 75th percentile), there was no statistically significant difference in relation to prejudice against TB patients (*p*-values =0.767). Regarding agreeableness, the median score was 5.00, with a range of 4.50–6.00 (25–75th percentile), it exhibited a significant negative correlation with prejudice against TB patients (*p*-values <0.001), indicating that students with higher desirability scores were less likely to harbor prejudice. For conscientiousness, the median score was 4.50, with a range of 3.50–5.00 (25–75th percentile), there was no statistically significant difference concerning prejudice against TB patients (*p*-values = 0.088). Regarding emotional stability, the median score was 4.50, with a range of 3.50–5.00 (25–75th percentile), there was a significant negative correlation with prejudice against TB patients (*p*-values < 0.001), suggesting that students with higher emotional stability scores were less likely to exhibit prejudice. In terms of openness to experiences, the median score of 4.50 with a range of 4.00–5.50 (25–75th percentile) was significantly and negatively correlated with prejudice against TB patients (*p*-values =0.004), indicating that students with higher openness scores were less likely to hold prejudice against TB patients (All *p*-values < 0.1) (Table 3).

**Table 3:** The distribution of the Big Five personality traits by prejudice stage.

Big Five personality traits from students		Total	Prejudice against TB patients		*P*	Yes	No
			*N* = 505	*N* = 289(57.23%)		*N* = 216(42.77%)
Extraversion	Median	4.00	4.00	4.00	0.767
	(P_25_–P_75_)	3.00–5.00	3.50–5.00	3.00–5.00	
Agreeableness	Median	5.00	5.00	5.50	**<0.001**
	(P_25_–P_75_)	4.50–6.00	4.00–5.50	4.50–6.00	
Conscientiousness	Median	4.50	4.50	4.50	**0.088**
	(P_25_–P_75_)	3.50–5.00	3.50–5.00	3.50–5.50	
Emotional stability	Median	4.50	4.00	4.50	**<0.001**
	(P_25_–P_75_)	3.50–5.00	3.50–5.00	4–5.50	
Openness to experiences	Median	4.50	4.50	4.50	**0.004**
	(P_25_–P_75_)	4.00–5.50	4.00–5.00	4.00–5.50	

P25-P75: 25th to 75th percentile; TB: Tuberculosis; Some *p*-values are highlighted in bold because they are less than 0.1.

### 3.3 The relationship between fear of TB/TB patients, knowledge of TB and prejudice against TB patients

Almost two-thirds (62.57%) of the medical students included in the study were fearful of TB/TB patients and almost half (45.54%) had poor knowledge of TB transmission. The univariate analysis showed that medical students who have a fear of TB/TB patients and a poor knowledge of TB transmission are more likely to have a prejudice against TB patients (All *p*-values < 0.1) (Table 4).

**Table 4:** TB knowledge, prejudice and fear in medical students.

Variable	Student		Prejudice against TB patients		*P*
	*N*	%	Yes (%)	No (%)	
Fear of TB patients					**<0.001**
Yes	316	37.53	232 (45.94)	84 (16.63)	
No	189	62.57	57 (11.29)	132 (26.14)	
TB knowledge					**0.003**
Good	275	54.46	141 (27.92)	134 (26.35)	
Poor	230	45.54	148 (29.31)	82 (16.24)	

TB: Tuberculosis; Some *p*-values are highlighted in bold because they are less than 0.1.

### 3.4 Multiple logistic regression analysis of independent influences on medical students’ prejudice against TB patients

According to the results of the multivariate logistic regression analysis, it was found that medical students who did not receive TB health education were 2.12 times (95% CI:1.35–3.32) more likely to hold prejudices against TB patients compared to those who received such education. Medical students who did not know a person with TB were 2.52 times (95% CI:1.39–4.56) more likely to hold prejudices against TB patients compared to those who knew TB patients. Additionally, medical students who fear TB/TB patients were 6.79 times (95% CI:4.36–10.56) more likely to hold prejudices against them compared to those who did not fear TB. Furthermore, the study revealed that residing in rural areas (OR: 0.60, 95% CI:0.38–0.95), higher levels of agreeableness (OR: 0.68, 95% CI:0.54–0.85) and emotional stability (OR: 0.80, 95% CI:0.65–0.99), and having a better understanding of TB knowledge (OR: 0.58, 95% CI:0.38–0.89) were significant protective factors associated with a lower likelihood of holding prejudices against TB patients (All *p*-values<0.05) (Table 5).

**Table 5:** Multiple logistic regression model to determine the factors associated with TB-related prejudice.

Variable	OR	95%CI		*P*
		Lower	Upper	
Whether you received TB health education? (RC:Yes)				
No	2.12	1.35	3.32	**0.001**
Know of a TB patient (RC:Yes)				
No	2.52	1.39	4.56	**0.002**
Birthplace (RC:Urban area)				
Rural area	0.60	0.38	0.95	**0.031**
Agreeableness	0.68	0.54	0.85	**0.001**
Emotional stability	0.80	0.65	0.99	**0.038**
Openness to experiences	0.83	0.68	1.02	0.070
Conscientiousness	1.12	0.91	1.36	0.290
Fear of TB/TB patients (RC:No)				
Yes	6.79	4.36	10.56	**<0.001**
TB knowledge (RC:Poor)				
Good	0.58	0.38	0.89	**0.014**

CI, Confidence interval; RC, Reference category; TB: Tuberculosis; Some *p*-values are highlighted in bold because they are less than 0.05.

## 4 Discussion

This study aimed to determine the factors influencing medical students’ prejudice toward patients with TB. More than half of the medical students in this study (57.23%) had prejudiced attitudes toward TB patients, lower than a study conducted in China on public stigma against TB patients, which showed about 71.9% had stigmatizing attitudes toward TB patients (49). Compared to the rest of the international, with results similar to those of a study conducted in Ethiopia (56%) (21), higher than India (51%) (16), and lower than in The Gambia (77%) (50). This difference may be related to the study design and regional context. Due to the high rate of prejudice against TB patients among medical students, interventions to reduce the prejudice of medical students against TB patients are necessary.

In our study that TB health education may reduce medical students’ prejudice against TB patients. This is understandable, students who had received TB health education tended to have a more accurate understanding of TB and therefore their prejudice against TB patients may be reduced. A study in Malaysia illustrated once again the importance of received TB health education improving attitudes toward TB patients (51). Moreover, this study found that nearly half of the medical students (45.54%) had poor knowledge of TB transmission. Medical students who were well informed about TB transmission were more likely to be free of prejudice toward TB patients. Perhaps as scholars such as Royce have mentioned, those with more knowledge, especially about infectiousness, were considered less likely to avoid or have negative attitudes toward TB patients (52). This reminds us that more emphasis should be placed on knowledge about infectiousness in future TB health education to better improve medical students’ prejudice against TB patients.

Our study found that knowing a person with TB played an important role in reducing prejudice against people with TB. One possible explanation is that exposure experiences help people to understand the feelings and worldview of the stigmatized group, thereby increasing empathy for the stigmatized group and reducing prejudice (53). Studies have shown that people who receive information about TB from people with TB have less prejudice against people with TB (20). Similarly, other studies also found a significant gap between whether relatives have TB and their understanding of TB (54). These findings suggest via learning about the patients’ own experiences, for example, by watching films based on the experiences of TB patients or by involving successfully treated patients as trainers in TB health education may be an effective way of reaching out to arouse people’s empathy toward patients and improve attitudes toward TB patients (55–57). At present, the common forms of TB health education for university students in China are mainly classroom lectures and special lectures (58). This suggest an interactive session with TB patients (if they are willing to reveal their identity) could be added to future TB health education for students. Such interactive sessions most commonly involve asking a recovered TB patient who has experienced stigma to act as a trainer or speaker, Talk about their own experiences of prejudice and its effects, etc., thus fostering empathy in medical students and reducing prejudice against patients.

Unexpectedly, the degree of prejudice against TB patients may vary by place of residence. Medical students who lived in rural areas were more likely to be less prejudiced against TB patients than those who lived in urban areas. Similar results were found in a study in Nigeria (59). In China, the incidence of TB tends to be higher in rural areas than in urban areas (60), and TB patients are more common in rural than in urban areas, meaning that rural residents are more likely to be exposed to TB patients and more aware of the disease, thus making them less prejudiced against TB patients than urban residents. Consequently, when developing interventions related to TB prejudice, it could be beneficial to concentrate on those medical students whose families reside in urban regions.

Fear of TB/TB patients was considered to be an important factor associated with prejudice against TB patients (20). Our analysis found that medical students who fear TB/TB patients are more likely to hold prejudicial attitudes toward TB patients. Previous studies have shown that medical staff’s fear of TB/TB patients leads them to maintain a certain distance and bias toward patients (18). Excessive fear of TB patients can lead health workers to adopt protective measures when dealing with TB patients, far outpacing the actual health risks of TB patients and increasing the stigma of TB patients (61, 62). Given the close correlation between prejudice and fear, there is an urgent need for an effective set of interventions to reduce fear of the disease, and more attention should be paid to medical students who may fear TB/TB patients.

In terms of the Big Five personality traits, this study shows that medical students who have more Emotional Stability and Agreeableness are more likely to be less prejudiced against people with TB. Those with a personality bias toward agreeableness tended to be considerate, friendly, generous, helpful and willing to compromise their interests with others (63). On the other hand, lower Agreeableness is often hostile and distrustful of others, which may distance oneself from the perceived threat of illness (64). This may make more agreeable people have a positive attitude toward TB patients so that they are less likely to be biased against TB patients. People who are more emotionally stable usually have a clear personality trait of being calm, relaxed, content with themselves and inclined to be pro-social, caring for the welfare of others, and often less self-interested (65–67), and it is perhaps this personality trait that makes them less likely to have a prejudiced attitude toward people with TB. Nonetheless, there is no literature on the impact of different personality traits on the level of prejudice among TB patients, but a recent study found that people with a personality bias toward emotional stability and agreeableness held less negative attitudes toward people with mental illness (68), a finding similar to our study. Brown suggests that using personality traits as a screening tool for healthcare providers may alleviate stigma in TB patients (69). Integrating personality modification interventions into educational programs for medical students might improve the attitude to TB patients. Future research still needs to explore personality trait-based interventions, which are important to improve medical students’ attitudes toward TB patients.

There are several limitations in the current study that must be mentioned. Firstly, this study is cross-sectional, which limits the establishment of causal relationships between variables, that a longitudinal study is needed. Secondly, we only conducted a quantitative analysis and not a qualitative one. Thirdly, other variables, such as the literacy level of the parents of medical students and the economic status of their families, may also influence medical students’ prejudice against TB patients, which was not addressed in our study. Finally, this study was conducted in only one medical school within Dalian. Due to regional differences in background, the results of the study cannot be generalized to other areas, and the results obtained are only representative of areas with the same conditions. Future research should include expanding the population, enriching the survey methodology, adding influencing factors, and different regions.

## 5 Conclusion

This study aimed to explore the current status of medical students’ prejudice against TB patients and its associated predictors. Our study found that medical students who are more agreeable and emotionally stable, have a good knowledge of TB transmission, and have a rural home are more likely to be less prejudiced against TB patients. However, medical students who had not received health education about TB, did not know a person with TB, and had a fear of TB/TB patients were more likely to have prejudiced attitudes toward TB patients. Consequently, interventions to reduce medical students’ prejudice against TB patients should allocate more resources to medical students whose families live in urban areas, who have not received TB health education, who do not know people with TB and who have a fear of TB/TB patients. Besides, the importance of medical students’ agreeableness and emotional stability, and their role in receiving TB health education and acquiring knowledge about TB transmission, should be emphasized to reduce medical students’ prejudice against TB patients. Medical students, who as a reserve of health workers, when seeing people with TB as ‘normal’, have the potential to change the public perception of the disease and improve public attitudes toward people with TB in the future.

## Data availability statement

The raw data supporting the conclusions of this article will be made available by the authors, without undue reservation.

## Ethics statement

The Ethical Committee of Dalian Medical University approved this study. Before the investigation, the students were informed of the purpose of the study and the presentation of the results, and they were assured that their personal information would not be disclosed. All students signed informed consent forms in our study.

## Author contributions

YaY: Writing – original draft, Writing – review & editing. MS: Writing – review & editing. XC: Writing – review & editing. YP: Writing – review & editing. JL: Writing – review & editing. YiY: Writing – review & editing. XD: Writing – review & editing. LZ: Writing – review & editing.
